# Supplementary material for: Revealing schoolchildren’s key situations in the use of digital media inside and outside school: A media diary study
Source: PLoS One. 2024 Dec 31;19(12):e0316567. doi: 10.1371/journal.pone.0316567 (PMC11687912; doi:10.1371/journal.pone.0316567)
Supplement: S1 Table — (DOCX) [file pone.0316567.s001.docx]

| **Child No.** | **Extraversion** | **Neuroticism** | **Openness** | **Agreeableness** | **Conscientiousness** |
| --- | --- | --- | --- | --- | --- |
| 1 | 2.67 | 3.60 | 3.86 | 3.20 | 3.67 |
| 2 | 4.67 | 3.40 | 4.43 | 3.80 | 3.67 |
| 3 | 2.33 | 2.40 | 3.43 | 4.00 | 3.67 |
| 4 | 1.67 | 3.80 | 3.71 | 4.00 | 4.00 |
| 5 | 3.67 | 3.20 | 3.86 | 3.60 | 3.17 |
| 6 | 5.00 | 2.40 | 4.14 | 4.80 | 3.67 |
| 7 | 4.00 | 2.40 | 4.00 | 3.80 | 4.17 |
| 8 | 5.00 | 1.00 | 4.57 | 4.60 | 4.67 |
| 9 | 1.33 | 3.60 | 4.00 | 4.40 | 2.00 |
| 10 | 2.67 | 3.00 | 3.43 | 3.00 | 3.83 |
| 11 | 1.00 | 4.00 | 3.00 | 4.20 | 4.17 |
| 12 | 4.33 | 2.20 | 3.86 | 4.00 | 3.50 |
| 13 | 3.33 | 2.00 | 4.29 | 4.60 | 4.50 |
| 14 | 3.67 | 2.40 | 3.71 | 4.20 | 4.00 |
| 15 | 2.33 | 3.20 | 3.57 | 4.60 | 4.17 |
| 16 | 4.33 | 2.20 | 4.00 | 3.80 | 3.17 |
| 17 | 2.00 | 4.20 | 3.43 | 2.00 | 2.33 |
| 18 | 1.00 | 3.80 | 3.43 | 3.00 | 3.33 |
| 19 | 2.00 | 4.00 | 4.00 | 3.80 | 3.50 |
| 20 | 5.00 | 1.80 | 4.29 | 3.80 | 4.67 |
| 21 | 2.00 | 3.00 | 3.14 | 3.60 | 3.33 |
| 22 | 1.00 | 4.00 | 4.57 | 4.20 | 4.50 |
| 23 | 2.67 | 2.80 | 4.57 | 4.20 | 3.33 |
| 24 | 2.67 | 3.00 | 3.29 | 4.00 | 2.50 |
| 25 | 4.33 | 2.60 | 3.43 | 4.00 | 4.00 |
| 26 | 3.33 | 3.20 | 3.14 | 3.60 | 3.33 |
| 27 | 3.67 | 1.60 | 2.57 | 3.40 | 3.67 |
| 28 | 1.33 | 2.40 | 2.71 | 3.80 | 3.67 |
| 29 | 2.00 | 3.40 | 4.71 | 2.80 | 3.67 |
| 30 | 3.00 | 2.60 | 3.71 | 3.20 | 4.00 |
| 31 | 4.67 | 2.80 | 2.71 | 4.00 | 3.33 |
| 32 | 3.33 | 2.00 | 3.43 | 4.00 | 3.83 |
| 33 | 4.33 | 2.60 | 4.71 | 5.00 | 2.00 |
| 34 | 2.33 | 3.60 | 3.86 | 2.40 | 2.33 |
| 35 | 4.67 | 2.40 | 2.71 | 2.60 | 3.50 |
| 36 | 5.00 | 2.00 | 4.57 | 4.20 | 4.33 |
| 37 | 5.00 | 2.80 | 3.29 | 4.00 | 3.33 |
| 38 | 3.33 | 2.60 | 3.43 | 3.40 | 3.83 |
| 39 | 4.00 | 3.40 | 4.14 | 4.00 | 2.83 |
| 40 | 2.67 | 2.80 | 3.14 | 4.20 | 2.83 |
| 41 | 4.67 | 1.20 | 4.00 | 4.40 | 4.83 |
| 42 | 2.00 | 4.00 | 2.71 | 2.20 | 2.50 |
| 43 | 4.33 | 2.00 | 4.43 | 4.60 | 4.00 |
| 44 | 5.00 | 3.00 | 3.86 | 4.20 | 3.50 |
| 45 | 4.00 | 2.20 | 3.29 | 3.80 | 3.50 |
| 46 | 3.33 | 2.80 | 4.71 | 3.80 | 4.33 |
| 47 | 1.00 | 4.80 | 3.71 | 3.80 | 2.33 |
| 48 | 5.00 | 2.80 | 2.43 | 4.80 | 3.33 |
| 49 | 5.00 | 3.60 | 3.71 | 4.00 | 2.50 |

**S1 Table.** Expression of the Big Five traits of all school children.
