# Supplementary material for: Revealing schoolchildren’s key situations in the use of digital media inside and outside school: A media diary study
Source: PLoS One. 2024 Dec 31;19(12):e0316567. doi: 10.1371/journal.pone.0316567 (PMC11687912; doi:10.1371/journal.pone.0316567)
Supplement: Attachment — Submitted filename: Response to reviewers.docx [file pone.0316567.s002.docx]

Thank you very much for taking the time to review our manuscript and for providing such constructive feedback. We have worked carefully to address each point raised by the reviewers in our revision, striving to respond thoughtfully to all comments. With feedback from five reviewers, there were naturally some diverging perspectives, and we made every effort to accommodate each of these views in a balanced manner. Below, we respond to each of the reviewers’ comments individually.

**Response to Reviewer 1:**

**What are the basic characteristics of the 8 schools where the research samples were collected? Are they of the same type, public or private schools? What is the scale of these schools, and how diverse is the student population in terms of individual differences?**

**1a)** We have provided more details about the schools in the „Materials and Methods“ and „Sample“ sections. The participating schools are public institutions located in urban and suburban areas. Additionally, we have indicated approximate schoolchildren population sizes for these schools. Please note that precise numbers are withheld to protect the anonymity of both schools and schoolchildren.
 **1b)** Regarding individual differences, we included the Big Five personality assessment from the initial survey. Results indicate that while schoolchildren exhibit expected individual variations, no notable patterns were observed at the school level. For an overview, we have created Supplementary File S1, which presents the Big Five trait distributions for all participating schoolchildren. We now discuss the influence of personality, socioeconomic status and cultural background on media usage in the „Nature of Key Situations“ section of the discussion.

**Can the sample size of 49 support the research conclusions? How were these 49 samples selected? Is there any reference material to back up the sample size?**

**2)** It is important to note that this study follows an exploratory approach, where no hypotheses were tested, and therefore, no a priori power analysis was conducted. Schoolchildren in this age group are generally a hard-to-reach group, which was further compounded by the fact that we conducted a repeated-measures study without any incentives. We cannot retrospectively expand the sample size. However, we have ensured that drop-out (23 schoolchildren) did not result in significant differences between participants and non-participants. Additionally, the sample’s Big Five mean scores fell within expected ranges (as noted in the „Sample“ section). There is no indication that our sample is unusual. In the „Limitations“ section, we now critically discuss sample size and referenced similar studies with comparable sample sizes.

**Considering the psychological characteristics of students aged 10-17, could these factors lead to different conclusions regarding media usage?**

**3)** We did not conduct statistical analyses based on age due to the majority of the sample falling within the 10-13 age range, with only a few older schoolchildren. Age was not a selection criterion; instead, we focused on schoolchildren from lower secondary school (grades 5 through 10). Nonetheless, we conducted a descriptive analysis of the data and found no notable patterns among the schoolchildren over 13 years old. We have clarified these age-related observations in the „The nature of key situations“ section and discuss findings in the context of previous studies.

**The article has sufficient data analysis in the Results section, but the academic depth of the analysis for the conclusions is lacking.**

**4)** In the revised version, we have ensured that the discussion closely aligns with the study’s findings. Any content beyond this has been clearly marked as such and primarily pertains to our recommendations for practice and policy. Reviewer 5’s sixth comment also suggests that these recommendations are welcome, though we aim to stay as close as possible to our results and limit speculative recommendations.

**The author's conclusion that „115 key situations occurred outside of school, while only 30 occurred in class“ likely reflects the genuine experiences of most students, as media use in schools is mainly for learning activities, while it gets more complex outside of education. My question is: since this conclusion seems very commonsensical, where does the significance and innovation of the research lie?**

**5)** As you noted, it may indeed be expected that more key situations occur in informal settings. This aligns with research indicating that media consumption is more frequent during leisure time. However, to our knowledge, no previous studies have explicitly examined key situations (or similar concepts) and their contextual settings. Thus, this finding is relevant for further research. Additionally, our study presents various findings, focusing not only on the settings but also on the content and circumstances of these key situations.

**Response to Reviewer 2:**

**The authors need to clarify specific details related to the study sample. For instance, it would be helpful to know the number of hours in the school day and whether there are any restrictions or regulations on students bringing digital devices (such as mobile phones or tablets) to school. There is a considerable difference between digital activities conducted inside and outside the classroom, and understanding the reasons for this variation would be beneficial.**

**1a)** Please refer to our second comment to Reviewer 1 regarding the specifications of the schools.

**1b)** The study took place at the end of 2021, when COVID-19 restrictions were gradually being eased. During this period, educational institutions were particularly open to new teaching formats and the use of digital media. Schools and higher education institutions adapted by integrating social media into their teaching strategies ([110]) and creating new opportunities for both students and educators to engage in innovative teaching and learning methods ([109]). The schools involved in our study demonstrated openness towards digital media, as they allowed the implementation of our research. However, whether and how digital media are used in classrooms in Germany is not standardized but depends on the individual teachers’ openness and creativity. This poses a fundamental challenge in terms of teaching media literacy. We now further discuss this aspect in the section „Setting in which key situations take place“ in the discussion.

**Regarding young students (10 years old) interacting in WhatsApp groups, how are children of this age engaging with such applications? Do parents monitor these groups?**

**2)** We indeed collected data on both hardware and software for each key situation. This aspect was previously omitted from the manuscript, as multiple entries were possible, and it was not feasible to reconstruct the specific role that a specific program played in each key situation. Up to five programs could be mentioned by the schoolchildren, and this option was often utilized. Nevertheless, we have taken the feedback into account and created two new graphs (see Fig. 4 and Fig. 5), with the former showing the digital media used, and Fig. 5 illustrating the ten most frequently used programs by the children. Additionally, we have included the hardware associated with a key situation in Fig. 1. We also added information to Fig. 1 on whether communication occurred regarding the situation or not, allowing us to determine whether, for instance, a key situation related to communication was discussed afterwards. These findings have been incorporated into the new „Hardware and Software“ subsection of the results and discussion sections. The role of parents in regulating access to WhatsApp was not part of this study, though there is interesting research on this topic.

**Regarding children participating in activities from Friday through Monday, I believe this period might favor recalling activities outside school. Tuesday through Thursday is a relatively long stretch for students to remember in-school activities. The authors should clarify why children could not record their weekly activities all the week’s days.**

**3)** Such a recency effect is indeed conceivable and cannot be ruled out. We have added this point to the limitations section. Nevertheless, our definition of a key situation includes the requirement that it is particularly important and, as such, is well remembered. If a situation is forgotten within a few days, it does not meet our definition. It was intentional that the survey focused on reflecting on the week as a whole, rather than being filled out immediately after the first situation. We outline these aspects in the revised manuscript.

**Lastly, focusing on the differences attributed to age in the results would enhance the discussion, as significant agerelated variations exist within the study sample.**

**4)** Regarding the differences between age groups, please refer to our third comment to Reviewer 1.

**Response to Reviewer 3:**

**It is mentioned in the text that it is a qualitative manuscript, however, it presents many quantitative analyses, so my recommendation would be to present it as a mixed concurrent research. It is true that the sample size is small, but it is mentioned in the limitations as an element to be taken into account.**

Thank you very much for the positive feedback. In fact, our study is not exclusively qualitative, but a mixed-methods approach with a focus on the qualitative facets of key situations. We have accordingly revised the wording throughout the paper. Furthermore, we have elaborated more on the discussion regarding the sample size (please see second comment to Reviewer 1).

**Response to Reviewer 4:**

**You should consider the journal's policy regarding the small sample size of the study, as only 49 schoolchildren were included in the analysis. Additionally, 22 schoolchildren were classified as dropouts (line 152), resulting in a response rate of approximately 70%. I recommend that the researchers address the high dropout rate, perhaps by analyzing demographic differences between the participating schoolchildren and dropouts (e.g., gender, school affiliation, grade).**

**1)** Please refer to our second comment to Reviewer 1, where we have provided a detailed explanation regarding the sample and dropout.

**In the first part of the introduction, I recommend adding a detailed definition of the components of 'digital media' for the various everyday purposes mentioned in line 36 (outside the classroom). This may include specifying the use of 'news media' through traditional online and social media platforms, other social interactions through social media, and additional digital media outlets such as office tools, search engines, video games, streaming entertainment, music, etc. If possible, relevant findings on different types of digital media could also enhance the analysis, especially by differentiating news media consumption from entertainment and documentation, which may offer a broader scope.**

**2a)** We have clarified the definition of „digital media“ in the introduction and provide examples of its use during leisure time.

**2b)** Concerning digital media (hardware/software), we have thoroughly reviewed the data and made several additions. First, we have updated Fig 1 to ensure that the type of digital medium associated with a key situation is visible. Additionally, we have added a new subsection, „Hardware and Software,“ in the Results chapter and we now also discuss these findings in detail in a dedicated subsection within the Discussion chapter.

**Did the study also include demographic data on the families-parents of the participating schoolchildren? Information such as parents' education level or family socioeconomic status could provide additional insights in interpreting the results.**

**3)** No, these demographic data and parental information were not collected. However, we have further clarified the description of the schools. We have also included the children’s Big Five scores as a supplementary file (S1).

**Regarding the finding that key situations varied significantly among schoolchildren at the individual level, the researchers suggest that differences in media usage could be linked to various motivational or personality factors (Table 4, after line 596). The researchers might also consider other factors that could influence differences in media usage, such as socioeconomic and cultural factors.**

**4)** We were pleased to incorporate this addition into the table. We briefly discuss this in the new subsection, „Hardware and Software.“ Since socioeconomic status and cultural factors were not measured, however, we cannot explore this topic further.

**I would suggest providing a more thorough definition of 'key situations' in the introduction section (line 43). The current definition is quite brief, and key situations are not limited to schoolchildren, and can also apply to adults.**

**5)** Yes, we have clarified the definition of „key situations“ in the introduction.

**Additionally, I noticed a typo error in line 42: "s is".**

**A grammatical remark: I noticed in line 28 "digital media" is presented in singular ("has"), however in other sentences it is presented in plural (for example in lines 34, 48, 342) – there should be uniformity, apparently in plural since the definition reflects several digital media outlets.**

**6 and 7)** Thank you for pointing out the spelling errors. We have corrected the specified points and rechecked the manuscript as thoroughly as possible.

**Response to Reviewer 5:**

**Title and Abstract: The title is clear and reflects the objective of the study; The abstract is well structured and covers the main points of the study. However, it is possible to simplify some sentences to make the reading more fluid, keeping the focus on the main findings.**

**1)** We have completely revised the abstract, aiming to simplify the sentences to improve readability and focus on the main findings.

**Introduction: The introduction provides a good background on children’s use of digital media in educational and noneducational contexts. However, I suggest including a clearer justification for the relevance of studying the difference in media use across these contexts.**

**2)** In the introduction, we have added a clearer explanation from the literature on why studying digital media in different contexts (formal vs. informal) is important.

**Methodology: The choice of the media diary as a qualitative method is appropriate, but it would be important to include more details on how the categories for qualitative analysis were developed; The relatively small number of participants (n=49) could be a limitation.**

**3a)** In the first section of the „Results,“ we have provided a more detailed description of the categorization process to make it clear how the qualitative analysis was conducted.

**3b)** Please refer to our second comment to Reviewer 1 regarding the sample size.

**Results: The presentation of the results is clear and well organized, especially regarding the analysis of emotional variation (valence and arousal). It could benefit from greater contextualization with previous studies on the emotional impact of media use by children and adolescents. Suggestion: for data visualization: Including graphs or infographics to illustrate key trends, such as the most used type of media.**

**4a)** We included previous studies on emotions and media consumption in children in the subchapter „Emotional experiences during key situations“ in the introduction. In addition, in the new subchapter „Hardware and Software“ in the discussion, we compare emotional experiences with digital media. However, we decided not to perform trend analyses aggregated across all children, as there was significant variation both within and between individuals which is one of the most important findings of this study.

**4b)** Regarding the most used digital media, we have created two new graphics. On the one hand, we show which digital media were used and how frequently (Fig. 4: Hardware), and on the other hand, we show the top 10 most frequently mentioned programs/websites (Fig. 5: Software). Additionally, we have expanded Fig. 1 by now displaying the digital media used for each key situation and addressing the differences in the discussion.

**Discussion is ok**

**5)** We have expanded and further substantiated the discussion thanks to the useful suggestions from the other reviewers.

**Conclusion: The conclusion summarizes the findings effectively, but could benefit from more direct suggestions for educational practice. For example, how schools can integrate these findings to improve classroom use of digital media in a balanced way with out-of-school use.**

**6)** In light of the fact that the study was primarily exploratory and yielded important insights, we can only provide suggestions for educational practice based on the findings presented in Table 4. However, we are unable to offer specific recommendations on how particular media types or content could be integrated into subject-specific teaching practices. That said, our findings can serve as a basis for further research, potentially informing evaluation studies aimed at developing detailed recommendations.
There have already been attempts to integrate media into subject-specific teaching, for example in mathematics or language education. However, these approaches are typically subject-specific and focus on the use of digital media within particular academic fields. In contrast, our study takes a broader approach: it examines the general use of digital media by schoolchildren and identifies themes and key situations that are relevant both in school and out-of-school contexts. These themes are not only important for the children’s personal and cognitive development, but also for promoting media literacy. A key challenge, however, is that digital literacy is not yet an independent subject in many countries, including Germany. Instead, it is treated as a cross-cutting theme, and its integration into individual subjects depends largely on the approach taken by the teacher. This makes it difficult to systematically and consistently embed media education into the curriculum, which in turn limits its potential to foster student motivation and individual development. We address this point briefly in the conclusion.

With all these changes, we believe we have substantially improved the manuscript. Thank you for your time and the opportunity to revise our manuscript. We look forward to your feedback.
